# Military Surgery in Paris

**Published:** 1848-06

**Authors:** 


### MILITARYSURGERY IN PARIS.

*Souvenirs of the 24th of February—the temporary Hospitals.*—On the 24th of February, while the lugubrious sound of the tocsin was mingled wiith the beating of drums and the reports of musketry, more than one hundred military officers of health stamped with impatience behind the gates of Val de Grâce, waiting only for an order, a word, to carry immediate succour to their brethren who fell. These orders, not to speak of commands, should come by means of the military intendants, but not an attendant appeared. These gentlemen had other things to think of, I suspect. I do not intend to throw blame on those justly-esteemed gentlemen; but passing over these, I will formally attack the principle. If the intendants cannot sufficiently watch over the accomplishment of measures so interesting to humanity, why confide this business to them?

In the meantime the impatience redoubles and increases, and already some of the surgeons provide themselves with the most indispensable materials, and proceed, either singly or in groups, to the quarters where their aid was required. The remainder still waiting. The firing and the cries increased. Shall they obey the voice of humanity, which cries "March!" or the barbarous and blind rule which commands inaction? Hesitation is impossible. The movable hospitals are regularly organized; every one wishes to form a part of them; they endeavour to obtain, as a favour, the privilege of following them. The administration readily complies with this unlooked-for appointment. The overseers of the military infirmaries are put at the disposal of the surgeons: they are furnished with litters, amputation instruments, and all the necessary materials for the first dressings. The mayorality of a district was appointed as the head-quarters of each moving hospital.

The first hospital, under the orders of Dr. Marney, the second under those of Dr. Radat, attended to the eleventh and twelfth wards; the fourth, with M. Cabasse at its head, whose name is so justly popular on account of his heroic conduct and rare devotion during his captivity in Africa. With his staff, he betook himself to the "Place de la Bastille," and there a great number of wounded experienced under his skilful hands the first ease to their pains.

We were at the head of the third ambulance, consisting of two assistants, MM. Laparay and Leroy, and ten pupils, all whose names we should mention, if we wished to name all who deserved it. Nine infirmary overseers followed us, carrying two litters. Our intention is not, God knows, to set our acts and movements before the eye of the reader. Such a pretension would not harmonize with the slight service which we rendered in the accomplishment of our duty; but our mingling with both the parties, and our contact with the population, both military and civil, in those moments of strife which exalt the passions to so high a degree, causes us to observe the attitude of each towards the medical body which had come forward to dress all their wounds.

The mayoralty of the tenth ward was our head-quarters. From Val de Grâce to the Rue de Grenella Saint Germain our march was one long ovation. The people understood by their admirable instinct that suffering makes all men equal, and that the surgeon, whenever a wounded man falls, hastens to succour him, without ever thinking if he marched under the flag of friends or enemies. "Vivent les Ecoles !—vive le Val de Grâce"—such were the cries with which we were everywhere greeted. A hundred fraternal hands pressed our hands ; hats were waved on the tops of sabres and guns. If one of us stumbled in climbing over a barricade, he was lifted up by the crowd, and carried and let down farther on in the clear road ; and the cries redoubled, and the hands gave us more numerous and closer embraces.

We were already more than paid for what we could do in our journey to stop the blood of this generous and noble people.

A little incident, notwithstanding, nearly proved fatal to us. One heroic apprentice-boy, as was seen before in 1830, carrying a gun taller than himself, threw a covetous glance on our swords, which he wished for his belt, which was hanging by his side without any arms. "Let us seize on their swords ; they do not want them !" cried he with a squeaking voice, with which was mingled other juvenile voices. Panurge's sheep are met with in all times and in every class. Affairs were positively threatening, but happily the hostile band was followed by a crowd which overwhelmed us with acclamations, and whose cheers drowned the cries of the first.

One of my companions was maltreated, and even was in positive danger. Whilst on the "Place de la Concorde," he was attending to a captain suffering from cerebral congestion ; he was assailed by three individuals who seized his sword, broke it, and threw away the fragments. The effervescence of the moment made them forget the inviolability with which our mission to all humanity surrounded us. Another man from the people ran up to him threatening him with death, and advancing his bayonet ; but at the sight of the instrument-case he stopped short, seized with remorse in the middle of his rage. Our companions' hands were seized by four of the common people, which seemed to say : "We wish you well : an instant's forgetfulness is very pardonable in those moments so full of great emotions and unexpected events."

Arrived at the municipal seat of the tenth ward, we were informed that there were no wounded in that quarter ; and notwithstanding the order which we had received to confine ourselves to that mayorality, we determined to seek to render ourselves useful in other places. They were fighting in the neighbourhood of the Carousal ; that was our place. In passing before the railing of the Tuileries we were not recognized ; some balls whistled over our heads and fell near us. But soon we were surrounded by a group of the armed people, who offered their protection. It became necessary to choose a place to set up our hospital in. We now heard only some scattered shots : our installation was made peaceably enough in the yard of a house situated nearly under the temporary wooden corridor which flanks the grand picture gallery. The wall of the court and the pannels of the gate appeared to us sufficient for the support of our wounded. We immediately set to work ; and some citizens were placed at the door as sentinels to prevent the crowd from coming in to interrupt us in our business. It was very necessary to hoist a signal which would make us known at a distance, and would command respect for the asylum of suffering. The red flag, the people's colour, would not suit us, for we required a standard essentially neutral and characteristic. A black silk apron, which was given us by a woman, was immediately tied to a long pole, and soon floated over our door. In the meantime a man, mounted on a ladder, and being provided with a bit of charcoal by way of brush, wrote "Ambulance," in large letters.

A horseman from the chateau was struck, not far from us, with several shots, which caused him to fall from his horse. I immediately sent a litter to take him up, and he was immediately brought into our hospital: our care was useless; a ball had cut through his carotid; he expired whilst coming in. But some of the people who followed the litter into the house discovered thirty or forty chasseurs standing by their horses in a stable. The people immediately set themselves to disarm them. Strife was about to commence even in our hospital—war to disturb the asylum of peace and of pain, and blood to flow in places set apart for the staunching of blood and closing of wounds! The place was no longer a place for us to remain; we quickly removed our hospital, and the closing of the door separated the two parties, and put an end to the commenced collision. We regained the Carousal with the bitter thought that we had brought about, although involuntarily, a combat in which our brethren were killing one another.

When we entered our hospital the Tuileries were surrounded by a compact body of troops of the line and of cavalry; all had been changed in that short time; the troops were retiring. For a moment the front of the chateau was bare, mournful, and silent; but presently the balcony door of the parlour of Marshals opened, all the windows are opened, and we saw the flag of the people and National Guard waving in the saloons. The strangest revolution had taken place before our eyes; the royal family had taken to flight, and the people were sovereign.

The firing ceased at the same time around the Tuileries; the only discharges which we heard was from the Palais Royal, where everything showed that the resistance was about to cease. Under these circumstances a central and fixed hospital semed to be the fittest for the purpose; it was necessary to multiply the succour by dividing the staff, so that the wounded might be collected from every part and transported to a proper place. The mayorality of the tenth ward again received us; there we divided into three sections, the first of which was under our orders, whilst the two others were confided to MM. Laparay and Leroy, whom I directed to the Palais Royal, protected by picquets of the National Guards, and accompanied by the overseers carrying the litters. It is to those two sections that I award the greatest part of the honour of that day; they dressed thirty or forty wounded, who were for the most part carried to the Hospital La Charité.

At the Palais Royal, the Orleans Gallery had been converted into a vast temporary hospital, where might be counted, for a long time, thirty or forty wounded, those whom they incessantly carried from the streets, which were the scene of combat, replacing those who were taken away in litters from the hospitals. The approaches were strictly guarded by some of the people and pupils of the Polytechnic School. Their vigilance guarded the passage against those for whom assistance was not necessary. The sumptuous canopies of the royal saloons served as beds for the wounded, and the head of the child of the people lay on rich damask cushions. The doctors in the civil service, the surgeons of the National Guard, and especially of Val de Grâce were there running from one wounded to another, but with the same care for all. A good many of them passed the day and the entire night in the hospital of the Orleans gallery.

The combatants of all parties who fell in the streets, in the neighbourhood of the Palais Royal, were carried to the Orleans gallery; many were taken up by the inhabitants of the neighboring houses who lavished on them the greatest care. The rich generally offered the cusions of their canopies; we have seen poor housekeepers tear up excellent shifts to supply us with bandages, and deprive themselves, in favour of the sufferers, of the only mattress which they possessed. It is sometimes true to say that the sight of blood stimulates the warlike instincts, and causes the hideous passions of vengeance to arise; it is necessary carefully to establish an exception in the circumstances where no longer a fallen enemy is to be dealt with, a conquered stranger, but a brother against whom unfortunate circumstances armed us for a moment. So the man from the people, the soldier of liberty, stretched out his hand to assist the soldier of the monarchy whom he had just conquered. "Honour to unfortunate courage," cried the emperor, whilst standing before the litter which carried the wounded of the conquered party; respect for all suffering, so thought the people in offering their shoulders to carry the wounded soldier, in addressing a few words to him, but which came from the heart, and expressed much in few words.

While MM. Laparay and Leroy laboured so actively, we gave our care to some men lying on the camp beds of the mayoralty guard. One was so strongly pressed by the crowd, that he was in danger of asphyxia; another, had received a ball in his leg; a third, had entombed in his stomach, unaccustomed to such things, some kidneys cooked with Madeira, which were prepared for the royal table, and had washed them down with sundry bottles of old Bordeaux. The excitement of the combat—excitement of the alcohol—you understand. As soon as he recovered from his collapse, he commenced brandishing a large wooden spoon, which he had held with tenderness against his breast. Six drops of ammonia caused him rapidly to come to himself; he got up, and cried, still hiccupping—"Vive la republique!" and went off with his precious trophy, which he called the king's spoon.

The evening came on, and there remained two men on the beds, one only of which required to be removed to Val de Grâce. We had neither litters nor overseers. A neighbour offered us a stretcher bed, which was kept spread open by two pieces of wood across, so as to form a litter, which could be easily carried. There remained to find the porters. I exposed my wounded man in the street on his bed, and I remained standing beside him. My mute appeal did not remain unanswered. Immediately the litter was taken up, and the train moved on, preceded by two National Guards: other armed men joined us on the way. Two women who followed us, sacrificed their handkerchiefs, which they folded into a cushion, and interposed between the shoulders of the porters and the wood of the stretcher. In the Rue de l'Ouest, the stretcher broke down in front of a shop; the proprietor immediately tore down a board from the front to assist us in mending it. In passing under a lamp I recognized amongst the porters my friend Moretti, sub-assistant surgeon, thus modestly making use of his physical powers after having made use of his scientifical.

During the 25th and 26th, the surgeons of Val de Grâce organized other hospitals which were sent to Neuilly, to Vincennes—everywhere in fact where they expected bloodshed. They also searched the civil hospitals to discover soldiers who might have been temporarily brought there, and to transport them to the military hospitals. The tranquillity which reigned since the 24th had not sufficiently reassured the overseers.

At the side of the military surgeons who were assisting the wounded, they saw others on foot and on horseback, conducting the popular groups, carrying the orders of the provisional government, overseeing the distributing of the provisions, and following the patrols destined to preserve the public peace. In these critical times the physician has two duties to perform, as a citizen and as a physician; Val de Grâce did not forget it for an instant. We only regret that the resemblance of the uniforms should cause confusion of the military with the pupils of the Polytechnic School. To be placed in public opinion, side by side, with this illustrious school, is, without doubt, a most enviable position; but notwithstanding we ought to keep our individuality perfect, when we have the sentiment of having conscientiously discharged our double duty, and when we have a colour brilliant enough to be put in competition with other colours.—*Dub. Med. Press, from Gaz. Médicale de Paris.*

